# High Vitamin D Concentrations Restore the Ability to Express LL37 by *M. tuberculosis*-Infected Human Macrophages

**DOI:** 10.3390/biom12020268

**Published:** 2022-02-07

**Authors:** María Teresa Herrera, Esmeralda Juárez, Silvia Guzmán-Beltrán, Martha Torres, Victor Adrián Luna-Morales, Leonardo Daniel Villalana-Alvarez, Yolanda González

**Affiliations:** 1 Department of Microbiology Research, National Institute for Respiratory Diseases, Ismael Cosío Villegas, Mexico City 14080, Mexico; teresa_herrera@iner.gob.mx (M.T.H.); ejuarez@iner.gob.mx (E.J.); sguzman@iner.gob.mx (S.G.-B.); 2 Biomedical Research Sub Direction, National Institute for Respiratory Diseases, Ismael Cosío Villegas, Mexico City 14080, Mexico; marthatorres98@yahoo.com; 3 Facultad de Ciencias, Universidad Nacional Autónoma de México, Mexico City 14080, Mexico; victorluna@ciencias.unam.mx (V.A.L.-M.); leodani16_va@hotmail.com (L.D.V.-A.)

**Keywords:** vitamin D, hyperglycemia, tuberculosis, *M. tuberculosis*, LL37

## Abstract

Vitamin D has an immunomodulatory function and is involved in eliminating pathogens. Vitamin D deficiencies reported in Type 2 diabetes mellitus (T2DM) patients make them more susceptible to developing tuberculosis (TB). The macrophages are the immune cells that control intracellular pathogens by producing the antimicrobial peptide cathelicidin-LL37. This pathway involves TLR activation by pathogens, vitamin D receptor (VDR) ligation, and the enzyme 1α-hydroxylase Cytochrome P450 Family 27 Subfamily B Member 1 (CYP27B1). However, it is not clear whether the biological actions of vitamin D are affected by high glucose concentrations. This study aimed to evaluate the vitamin D contribution in the expression of VDR and CYP27B1, involved in the conversion of an inactive to an active form of vitamin D in the infected macrophages using *M. tuberculosis* as an infection model. The expression of LL37 and the nucleus translocation of VDR were evaluated as the readout of the response of vitamin D and determined if those processes are affected by glucose concentrations. Macrophages from healthy donors were cultured under glucose concentrations of 5.5, 15, or 30 mM, stimulated with vitamin D in inactive (25(OH)D_3_) or active (1,25(OH)_2_D_3_) forms, and infected with *M. tuberculosis*. The vitamin D-dependent induction of LL37 and the expression of VDR and CYP27B1 genes were analyzed by qPCR, and VDR translocation was analyzed in nuclear protein extracts by ELISA. *M. tuberculosis* downregulated the expression of LL37 regardless of the glucose concentration, whereas VDR and CYP27B1 upregulated it regardless of the glucose concentration. After evaluating two concentrations of vitamin D, 1 nM or 1 μM, the high concentration (1 μM) was necessary to restore the induction of LL37 expression in *M. tuberculosis*-infected macrophages. High concentrations of the inactive form of vitamin D restore the infected macrophages’ ability to express LL37 regardless of the glucose concentration. This finding supports the idea that vitamin D administration in patients with T2DM could benefit TB control and prevention.

## 1. Introduction

Vitamin D plays a role in regulating immune system cells such as monocytes/macrophages. Vitamin D is partially sourced through the diet (about 20%), but the most significant amount (80%) is synthesized via several biochemical processes within the body [1,2]. In mammals, it is produced under ultraviolet light from certain provitamins, such as 7-dihydrocholesterol, in the skin. In the liver, it becomes biologically active after hydroxylation. Vitamin D is converted to 25-hydroxycolecalciferol (25(OH)D_3_), which then turns into the active form 1,25-dihydroxycolecalciferol (1,25(OH)_2_D_3_) through a 1α-hydroxylase enzyme Cytochrome P450 Family 27 Subfamily B Member 1 (CYP27B1) in the proximal renal tubules or within immune cells such as macrophages.

In the cytoplasm of macrophages, the active vitamin D binds to vitamin D receptors (VDR) and induces the expression of antimicrobial effectors such as cathelicidin (also known as LL37) [1,3,4,5,6]. LL37 is an amphipathic, alpha-helical antimicrobial peptide generated by serine protease (proteinase 3) cleavage of the C-terminal end of the protein hCAP18 [7]. LL37 preferentially interacts with negatively charged bacterial membranes, forming pores with detergent-like effects, and controls pulmonary diseases including pulmonary tuberculosis (TBP) by inducing autophagy [8,9,10,11].

Vitamin D improves innate immunity against *M. tuberculosis* in monocytes/macrophages treated with the active or inactive form of vitamin D (1,25(OH)_2_D_3_) in vitro [12]. The innate receptors, Toll-like receptors 2/1(TLR2/1), sense *M. tuberculosis*, and together with VDR and CYP27B1, are involved in vitamin D metabolism and induce antimycobacterial activity [5,12]. The pathogen-associated molecular patterns (PAMPs) from *M. tuberculosis,* sensed by TLR2/1, induce the expression of the CYP27B1 protein, which converts 25(OH)D_3_ into the active form (1,25(OH)_2_D_3_) [5,13]. Subsequently, 1,25(OH)_2_D_3_ enhances antimicrobial activities in macrophages in an autocrine manner. Then, 1,25(OH)_2_D_3_ binds the VDR and retinoid X receptor (RXR), forming a complex which is translocated to the nuclei and is essential for high-affinity DNA binding to cognate vitamin D response elements (VDREs) located in the regulatory regions of 1,25(OH)_2_D_3_ target genes to directly induce the transcription of antimicrobial peptides as LL37 [3].

Low levels of 25(OH)D_3_ increase the risk of type 2 diabetes mellitus (T2DM) [14], and in T2DM patients, whose vitamin D levels are low, vitamin D supplementation significantly increases its serum levels and reduces insulin resistance [15]. In addition, vitamin D deficiency is associated with a higher risk of TBP and progression to severe disease forms [16,17,18], and low levels of vitamin D are associated with reduced local LL37 expression in active pulmonary TB [19]. However, vitamin D use as a complementary treatment for TB remains uncertain because oral supplementation does not reduce the risk for TB infection or disease [6,20]. Several meta-analyses of clinical trials have evaluated the benefit of oral vitamin D supplementation to complement TB treatment with controversial results. [21,22,23,24,25]

People living with T2DM are more likely to develop active TB once infected and are more likely to have poor TB-treatment outcomes [26]. The association of lower vitamin D levels with hyperglycemia has been reported in T2DM and women with pre-diabetes [15,27]. However, the effect of glucose concentrations on the biological actions of vitamin D is not clear.

This study aimed to evaluate the vitamin D contribution in the expression of VDR and CYP27B1, involved in the conversion of the inactive to the active form of vitamin D in the infected macrophages. We evaluated the Vitamin D response in infected macrophages through the expression of LL37 and the nucleus translocation of VDR as the readout and determined if those processes are affected by glucose concentrations.

## 2. Materials and Methods

### 2.1. Isolation of Peripheral Blood Mononuclear Cells (PBMCs)

Buffy coats from healthy donors were obtained from the blood bank at the National Institute for Respiratory Diseases Ismael Cosío Villegas (INER) in Mexico City. The buffy coats were diluted 1:1 in culture medium RPMI-1640 (Lonza, Walkersville, MD, USA) and separated by centrifugation over a gradient of lymphocyte separation solution (Lonza). Peripheral blood mononuclear cells (PBMCs) were recovered, washed twice in culture medium, resuspended in supplemented medium (RPMI 1640 plus 2 mM L-glutamine) and adjusted to 1 × 10^6^ cells/mL. The viability of the PBMCs was determined by trypan blue exclusion, which was >95% in all experiments.

### 2.2. Monocyte Enrichment

The monocytes were isolated from PBMCs by positive selection using the MACS separation system (MACS, Miltenyi Biotech, Auburn, CA, USA), according to the manufacturer’s recommendations. Briefly, PBMCs were mixed with anti-human CD14 monoclonal antibody conjugated to magnetic beads and incubated at 4 °C for 15 min, followed by a wash with buffer (2 mM EDTA, 0.5% Bovine Serum Albumin in PBS 0.01 M, pH 7.2). After several elution steps in a magnetic column, the CD14 positive cells (monocytes) were obtained. The pure monocyte population was centrifuged and resuspended in supplemented medium containing 10% heat-inactivated Fetal Calf Serum (FCS, Hyclone, Logan, USA), counted, and assayed for viability by trypan blue exclusion. On average, viability was >98%.

### 2.3. Purity of Monocytes

The purity of the monocytes (MNs) was evaluated by flow cytometry. Briefly, the MNs were surface stained with anti-human CD14-PE antibodies (BD, San José, CA, USA) via incubation for 15 min at 4 °C in the dark, then the MNs were washed with buffer (PBS, 2% FCS, 0.01% Sodium azide), and centrifuged. Finally, the MNs were resuspended in the buffer and acquired 10,000 events in a flow cytometer FACSAria Fusion (BD, San Jose, CA, USA). From the dot plot Forward Scattered (FSC) vs. Side Scattered (SSC), the MNs population was analyzed using the Flow Jo software version 10.4.1. Mac OS X. The percentage of CD14 positive cells was >95% for every experiment.

### 2.4. Monocyte-Derived Macrophages (MDM) Preparation and Infection

We incubated 1 or 2.5 × 10^6^ monocytes/well on ultralow attachment 24-well culture plates (Corning, Costar, New York, NY, USA) at 37 °C, in 5% CO_2_ over five days in supplemented medium containing 10% FCS to let them differentiate into MDM (macrophages). The medium was then replaced by a culture medium containing 5.5 mM of glucose (model of clinical normoglycemia equivalent to 90 mg/dl of fast serum glucose), or 15 mM or 30 mM of glucose (model of clinical hyperglycemia equivalent to 280 and 540 mg/dl of fast serum glucose, respectively) and incubated for 24 h. The macrophages were infected with *M. tuberculosis* H37Ra at a multiplicity of infection (MOI) of 1 for one hour. After washing out the non-internalized mycobacteria, the cells were treated with 10 nM or 1 μM of the inactive (25(OH)D_3_) or active (1,25(OH)_2_D_3_) forms of vitamin D. The infected macrophages were cultured for an additional 24 h. Then, the supernatants were removed and the infected macrophages were lysed with RNA lysis buffer for total RNA extraction by column (Qiagen, Hilden, Germany).

### 2.5. RNA Extraction and Quantitative Real-Time PCR (qPCR)

Total RNA was purified using the RNeasy Mini Kit (Qiagen Co., Strasse, Germany), according to the supplier’s instructions, followed by cDNA synthesis using reverse transcription with the Super-Script First-Strand cDNA Synthesis System (Invitrogen, Carlsbad, CA, USA) in a thermocycler Verity (Applied Biosystems, Foster City, CA, USA). Then, the cDNA was used to evaluate gene expression by real-time qPCR using TaqMan pre-designed assays for LL37 (CAMP, Hs00189038_m1-FAM), VDR (Hs01045840_m1-FAM), CYP27B1 (Hs00168017_m1-FAM), and ribosomal RNA 18S (rRNA 18S-VIC) (Thermo-Applied Biosystem). Amplification was performed in a StepOne Plus Real-Time PCR System (Applied Biosystems), and the data were analyzed with the 7500 Fast System SDS software (Applied Biosystems). The expression level results for each gene were estimated by the comparative ∆∆CT method, using the medium as a calibrator, or reported as relative to 18S.

### 2.6. Nuclear Extracts Preparation

Afterwards, 2.5 × 10^6^ infected macrophages were recovered, centrifuged, and resuspended in medium. Nuclear extracts were obtained by rupturing plasma membranes, isolating the nuclei, washing, and further lysing the nuclei following the previously described method [28]. The nuclear extract protein was determined in the nuclear extracts using the Bradford method (BioRad, Hercules, USA), including Bovine Serum Albumin (BSA, Calbiochem, San Diego, CA, USA) as a standard curve. The integrity of the nuclei was evaluated before the lysis by flow cytometry staining with the probe 7-aminoactinomycin D (7AAD, Biolegend, San Diego, CA, USA) that stains nucleic acids. In addition, 10,000 events were acquired in a flow cytometer FACSAria Fusion (BD) and analyzed using Flow Jo software version 10.4.1. for Mac OS. The nuclei region was defined from the dot plot FSC vs. SSC, and 7AAD staining evaluated the nuclei purity.

### 2.7. Vitamin D Receptor (VDR) Quantification

The nuclear protein extracts were used for VDR nuclear translocation detection using a commercially available ELISA immunoassay (BlueGene, Atlanta, GA, USA), following the manufacturer’s instructions. The detection was determined spectrophotometrically at 450 nm wavelength in a plate reader (Thermo Fisher Scientific Inc., Waltham, MA, USA) and reported as pg/μg nuclear protein.

### 2.8. Statistical Analysis

Statistical analysis and graphics were performed using GraphPad Prism version 9.1.0 software (GraphPad, San Diego, CA, USA). We used Wilcoxon’s signed-rank test for the pairwise comparison of matched samples. We performed Friedman’s or Kruskal-Wallis’ ANOVA followed by Dunn’s post-test (matched or unmatched samples, respectively) for multiple comparisons. We performed Wilcoxon’s one-sample test for normalized data to establish differences from median values = 1. Categorical data were analyzed with Fisher’s exact test. The significance was set at *p* < 0.05.

## 3. Results

### 3.1. M. tuberculosis Downregulates LL37 Expression at High Glucose Concentrations

**Figure 1:**
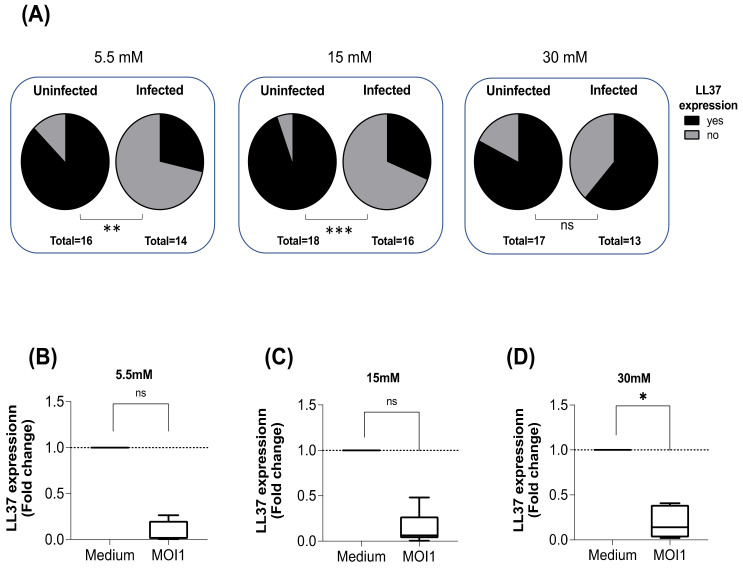
Effect of glucose and *M. tuberculosis* infection on LL37 expression. Macrophages were cultured for 24 h in different glucose concentrations (5.5, 15, or 30 mM) at 37 °C and 5% CO_2_ and then infected with *M. tuberculosis* H37Ra at MOI 1. After 24 h of incubation, the supernatants were removed, and the cells lysed for total RNA extraction, cDNA synthesis, and LL37 gene expression measurement by qPCR. Proportion of individuals expressing and unable to express LL37 (**A**), n = 16–18; ns: not significant, ** *p* < 0.01, *** *p* < 0.001, Fisher’s exact test for categorical variables. LL37 gene expression at 5.5 mM glucose (**B**), 15 mM glucose (**C**)**,** and 30 mM glucose; (**D**) depicted are box plots with median and quartiles. ns: not significant, * *p* < 0.01, Medium vs. *M. tuberculosis* (MOI 1), Wilcoxon test.

The expression of LL37 was investigated in human macrophages infected in vitro in the presence of 5.5, 15, and 30 mM of glucose. Glucose 5.5 mM was used as a normoglycemia, and 15 mM and 30 mM as hyperglycemia models [29]. Not all subjects expressed LL37 constitutively; 87% of subjects expressed LL37 in normoglycemia (Figure 1A), and the increase of glucose concentrations did not significantly modify the proportion of individuals expressing the antimicrobial peptide in uninfected macrophages. However, infection with *M. tuberculosis* downregulated macrophages’ LL37 expression regardless of the glucose concentration; the expression of LL37 was detected in 28, 31, and 61% of the subjects in 5.5, 15.5, and 30 mM, respectively (Figure 1A). Then, the fold change in LL37 expression was calculated in those individuals. A few subjects remained able to express LL37 (4 out of 12 at 5.5 mM glucose and 5 out of 12 at 15 mM glucose). Although the LL37 downregulation was remarkable, it did not reach statistical significance (Figure 1B,C). Moreover, at 30 mM of glucose, 8 out of 13 subjects expressed LL37 after infection, and it was significantly downregulated after *M. tuberculosis* infection (Figure 1D).

### 3.2. M. tuberculosis Upregulates VDR and CYP27B1 at Different Glucose Concentrations

**Figure 2:**
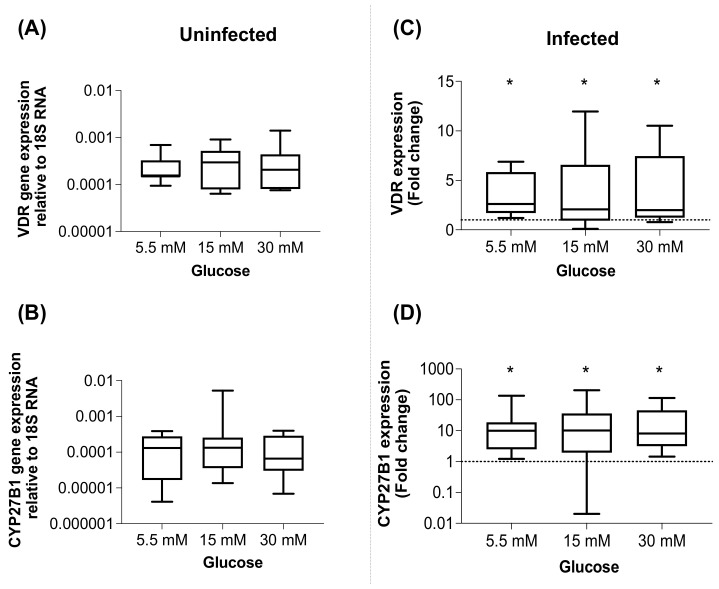
*M. tuberculosis* induces upregulation of VDR and CYP27B1. Macrophages were cultured for 24 h under different glucose concentrations (5.5, 15, or 30 mM) (**A**,**B**) and then infected with *M. tuberculosis* H37Ra at MOI 1 (**C**,**D**). After 24 h of additional incubation, the supernatants were removed and the macrophages lysed for total RNA extraction used for cDNA synthesis and VDR and CYP27B1 gene expression analyzed by qPCR. Uninfected macrophage gene expression is reported relative to 5.5 mM of glucose. Gene expression of the infected macrophages is reported relative to uninfected macrophages. Depicted are box plots with median and quartiles. * *p* < 0.01, 5.5 mM, 15 mM or 30 mM vs. Medium, Wilcoxon test.

The VDR and the enzyme responsible for metabolizing the inactive into the active form of vitamin D (CYP27B1) are critical elements of the vitamin D-dependent antimicrobial actions induced by pathogens. The effect of *M. tuberculosis* infection on the expression of the VDR and CYP27B1 was investigated in macrophages cultured in high glucose to identify the pathway of downregulation of LL37 expression by *M. tuberculosis*. The glucose concentrations (5.5, 15, and 30 mM) in the medium did not modify the expression of VDR and CYP27B1 relative to their expression under 5.5 mM in uninfected macrophages (Figure 2A,B). Furthermore, the infection significantly upregulated the expression of the VDR and CYP27B1 relative to uninfected macrophages, and this overexpression was not affected by high glucose concentrations (Figure 2C,D). Then, the effect of adding an active or inactive form of vitamin D on the expression of VDR and CYP27B1 genes at high glucose concentrations was investigated. Neither inactive 25(OH)D_3_ nor active 1,25(OH)2D_3_ vitamin D modified the VDR or CYP27B1 expression in infected macrophages at 5.5 and 30 mM, but with 15 mM, the inactive vitamin D caused a significant reduction in CYP27B1 expression. (Supplementary Figure S1A,B). In uninfected macrophages, neither of the vitamin D forms modified VDR or CYP27B1 expression (Supplementary Figure S2A,B).

### 3.3. The Inactive Form of Vitamin D (25(OH)D_3_) Restores the Expression of LL37 by Macrophages Infected with M. tuberculosis

**Figure 3:**
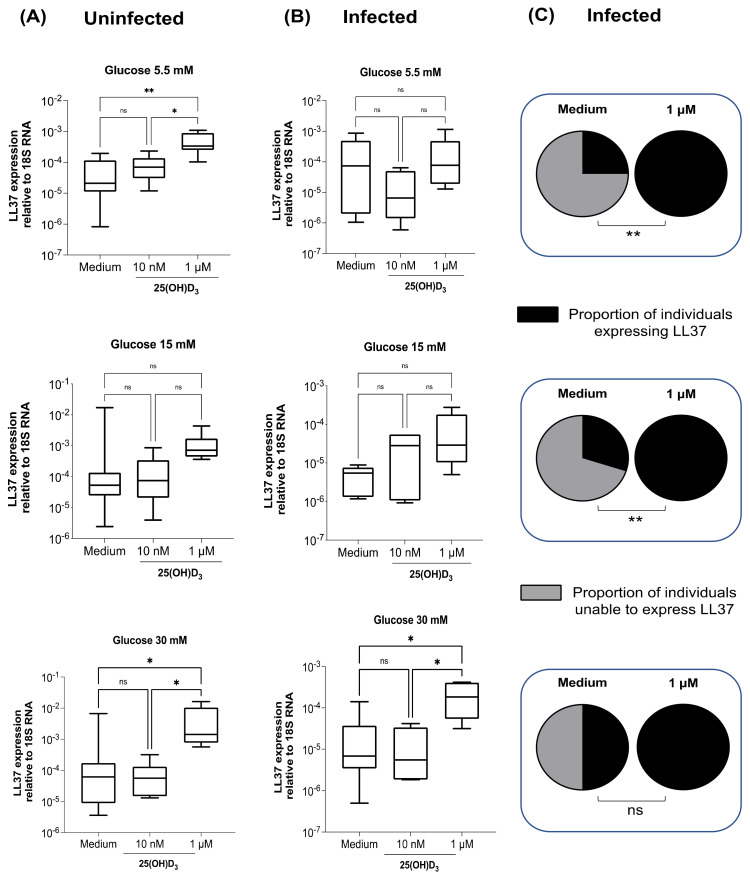
25(OH)D_3_ increases LL37 gene expression in infected macrophages. Macrophages were cultured for 24 h in different glucose concentrations (5.5, 15, or 30 mM) (**A**), or infected with *M. tuberculosis* (MOI 1), (**B**) supplemented with 25OHVitD (10 nM or 1 μM), and incubated for an additional 24 h. The macrophages were lysed, and the total RNA was purified and used for cDNA synthesis. LL37 gene expression was measured by qPCR and reported as relative to 18sRNA. Depicted are box plots with medians and quartiles. ns: not significant, * *p* < 0.01, ** *p* < 0.001. Medium vs. 25(OH)D_3_ (10 nM or 1 μM), Kruskal-Wallis’ nonparametric ANOVA followed by Dunn’s post-test. Proportion of individuals expressing and unable to express LL37 (**C**); ** *p* < 0.001, Fisher’s exact test for categorical variables.

Since the VDR and CYP27B1 are unmodified by glucose concentration, and LL37 upregulation by vitamin D in macrophages has been previously described [5,30], the effect of the addition of the inactive form of vitamin D (25(OH)D_3_) to upregulate LL37 expression was evaluated in infected macrophages at high glucose concentrations. The low dose (10 nM) did not increase the expression of LL37 in uninfected macrophages regardless of the glucose concentration (Figure 3A); however, a high dose of (1 μM) of 25(OH)D_3_ significantly upregulated LL37 expression at 5.5 mM and 30 mM of glucose. Because *M. tuberculosis* downregulates LL37 expression in macrophages, the ability of 25(OH)D_3_ to restore its expression was evaluated. The low dose of 25(OH)D_3_ (10 nM) was insufficient to increase the percentage of subjects expressing LL37 during infection (Figure 3B), but the high dose (1 μM) significantly increased LL37 expression at 30 mM of glucose. The lack of response with 25(OH)D_3_ 10 nM prompted the use of the active form 1,25(OH)_2_D_3_ at the same concentrations (10 nM and 1μM). The same phenomenon was observed with both 25(OH)D_3_ and 1,25(OH)_2_D_3_ in uninfected macrophages (Supplementary Figure S3A–C). Thus, 1 μM is the best concentration to be used in further investigation. Moreover, in additional assays, upregulation of LL37 expression was observed, with the addition of 1 μM of either form of vitamin D regardless of the infection and the glucose concentration (Supplementary Figure S4A,B).

### 3.4. Vitamin D’s Inactive Form 25(OH)D_3_ Increases the VDR Nuclear Translocation in Infected Macrophages

**Figure 4:**
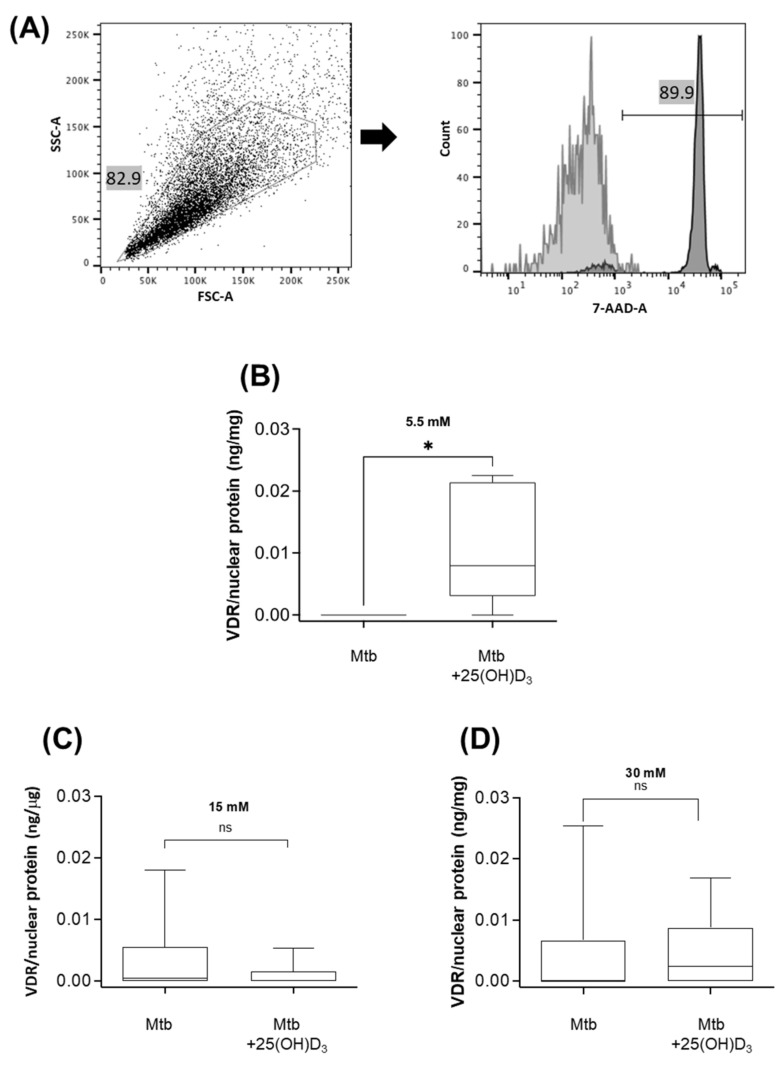
Effect of 25(OH)D_3_ on VDR nuclear translocation in macrophages infected with *M. tuberculosis*. Macrophages were cultured for 24 h in different glucose concentrations (5.5, 15, or 30 mM), infected with *M. tuberculosis* (MOI 1), supplemented with 25(OH)D_3_ (1 μM), and incubated for an additional 24 h. Nuclei were isolated and stained with 7-AAD for nuclei integrity verification by flow cytometry. Nuclei were gated according to their light scattering pattern. The histogram in light gray depicts the unstained sample (negative control), the histogram in dark gray depicts the 7-AAD stained sample of a representative experiment; 89.9% of the events were 7-AAD+ nuclei (**A**). Nuclear extracts were obtained from nuclei, and the vitamin D receptor (VDR) nuclear translocation was detected by ELISA. Box plots with individual results of cells in 5.5 mM (**B**), 15 mM (**C**), and 30 mM (**D**) glucose concentrations are depicted. ns: not significant, * *p* < 0.05, *M. tuberculosis* vs. *M. tuberculosis* + 25(OH)D_3_, one-tailed Wilcoxon test.

To evaluate if LL37 restoration is a vitamin D-induced mechanism, the nuclear translocation 1,25(OH)_2_D_3_-VDR-RXR complex was evaluated. Macrophages metabolize 25(OH)D_3_ into the 1,25(OH)_2_D_3_ form, which binds the VDR-RXR complex and is translocated to the nucleus to exert transcriptional activities [31]. The nuclear translocation of VDR was investigated under 25(OH)D_3_ (1 μM) and *M. tuberculosis* infection. After 24 h of vitamin D treatment, the nuclei were isolated (Figure 4A) and the VDR nuclear translocation was measured in infected macrophages treated with 25(OH)D_3_ and cultured with different glucose concentrations (5.5, 15, or 30 mM) (Figure 4B–D). The VDR nuclear translocation was observed in infected macrophages treated with 25(OH)D_3_ and cultured in 5.5 mM glucose.

## 4. Discussion

**Figure 5:**
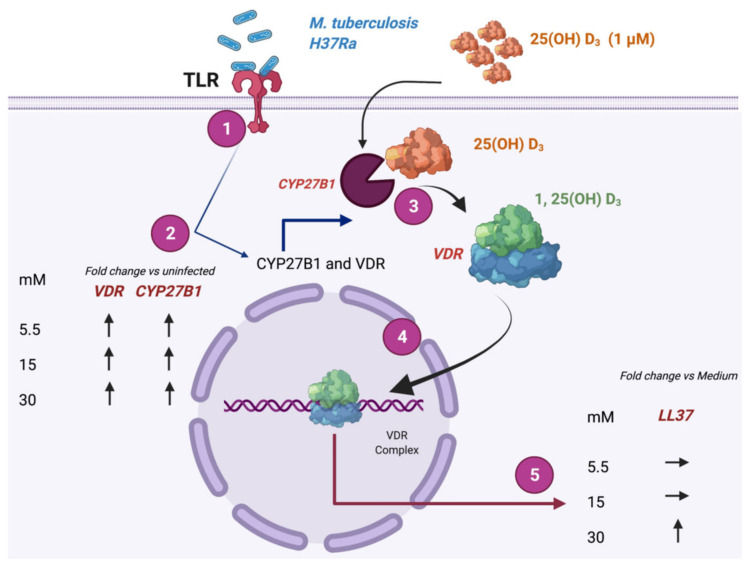
Overview of the effect of high glucose concentrations on LL37 expression dependent on Vitamin D. 1. Recognition of *M. tuberculosis* by Toll-like receptors (TLRs). 2. Induction of NF-kB activation and induction of CYP27B1 and vitamin D receptor (VDR) enzymes. 3. Conversion of vitamin D 25(OH)D_3_ into its active form 1, 25(OH)D_3_ by CYP27B1 and recognition by the VDR. 4. translocation of the VDR complex to the nucleus. 5. Induction of LL37 expression. Effect of high glucose on gene expression. Created with BioRender.com (accessed on 21 December 2021, QZ23I2DPQW).

In this study, we observed the overexpression of vitamin D metabolism-dependent elements such as CYP27B1 and VDR induced by the non-virulent strain of *M. tuberculosis* (H37Ra), and the in vivo conversion of vitamin D to its active form through the action of CYP27B1, with the subsequent overexpression of LL37 as part of the genes induced by the Vitamin D-VDR complex nuclear translocation in hyperglycemic conditions (Figure 5).

The virulent strain of *M. tuberculosis* is known to suppress the expression of LL37 in macrophages [32]. In this study, the avirulent strain exhibited the same effect, suppressing LL37 expression in human macrophages, probably because the avirulent strain conserves genes involved in survival and persistence, similarly to the virulent strains [33]. Hyperglycemia has been associated with the macrophages’ inability to induce antimicrobial response towards *M. tuberculosis* [34,35,36,37], which corresponds to the diminished ability of T2DM patients to upregulate the expression of LL37 [38]. Similarly, we found that the high glucose concentrations, resembling transient hyperglycemia, interfere with the macrophages’ ability to upregulate the expression of antimicrobial peptide LL37 after *M. tuberculosis* H37Ra infection.

One of the pathways for the induction of antimicrobial peptides such as LL37 is vitamin D-dependent. Recognition of *M. tuberculosis* ligands by TLRs induces the expression of CYP27B1, a 25-hydroxyvitamin D3 1-α-hydroxylase that catalyzes the conversion of inactive vitamin D (25(OH)D_3_) into the bioactive form of vitamin D (1, 25 (OH)2D_3_). The complex formed by 1, 25 (OH)2D_3_ and the VDR is translocated to the nucleus, with the subsequent transcriptional induction of LL37 [5,12,39,40,41,42,43]. Here, the overexpression of VDR and CYP27B1 induced by *M. tuberculosis* infection was not affected by high glucose concentrations, suggesting a possibility for vitamin D supplementation in patients with diabetes mellitus and TB.

Our results also demonstrated that nuclear translocation of vitamin D occurs in monocytes stimulated with 25(OH)D_3_ and infected with *M. tuberculosis*. The VDR remained in the nucleus after 24 h of treatment in normoglycemic conditions, but this did not occur in hyperglycemia. Previous reports using a promyeloblast cell line showed that the maximum translocation of VDR is reached after three hours and is still present after 24 h in normoglycemia [44]. It is possible that translocation kinetics are different in cells cultured in high glucose concentrations, which modify the effect of 1,25 (OH)D_3_ to stimulate VDR transactivation activity or retard VDR degradation [45]. Despite the absence of nuclear VDR, the upregulation of LL37 expression occurred at high glucose concentrations. In addition to the VDR complex, other transcription factors may be responsible for this biological effect, such as the signal transducer, activator of transcription 3 (STAT3) and hypoxia-inducible factor 1-alpha (HIF-1α), which also have binding sites on the LL37 promoter [46]. Notably, our data suggest that supplementation with vitamin D could similarly enhance LL37 induction in patients with or without T2DM [47].

We found that active or inactive vitamin D increases the expression of LL37 at 1 μM, but the inactive form has a higher circulating half-life and is more stable than the active form, which is relevant for treatment decisions [48,49]. However, it has been reported that an excess of vitamin D (hypervitaminosis D) is caused by prolonged consumption (months) of vitamin D mega-doses, leading to serious health consequences [50]. Clinical trials that use high concentrations of vitamin D (300 IU equivalent to 72 μg) for a short term achieve better results than low concentrations regularly [47]. It is possible that, due to the high concentrations of vitamin D required to observe an effect on the induction of vitamin D-dependent genes in macrophages, higher concentrations than those evaluated so far may be required to observe an effect as an adjunct to anti-TB therapy.

Several questions remain to be elucidated, such as the identification of the VDR-vitamin D complex in the nucleus, the mechanisms of vitamin D regulation, the role of the 24-hydroxylase (CYP24A1) that breaks down the active form of vitamin D to an inactive form in patients with TB and T2DM, and oral supplementation with higher concentrations of 25(OH)D_3_ than those evaluated so far.

## 5. Conclusions

A high concentration of 25(OH)D_3_ restored the ability of macrophages infected with *M. tuberculosis* to express cathelicidin LL37, regardless of glucose concentration.

## Data Availability

All data reported are included and represented in the manuscript.

## Supplementary Materials

The following supporting information can be downloaded at: www.mdpi.com/article/10.3390/biom12020268/s1, Figure S1: Effect of vitamin D on VDR and CYP27B1 gene expression in infected macrophages; Figure S2: Effect of vitamin D on VDR and CYP27B1 gene expression in uninfected macrophages; Figure S3: 1,25(OH)2D3 increases LL37 gene expression in infected macrophages; Figure S4: 25(OH)D3 and 1,25(OH)2D3 equally increase LL37 gene expression in macrophages independently of the glucose 30 concentration.
