# Supplementary material for: High Vitamin D Concentrations Restore the Ability to Express LL37 by M. tuberculosis-Infected Human Macrophages
Source: Biomolecules. 2022 Feb 7;12(2):268. doi: 10.3390/biom12020268 (PMC8961537; doi:10.3390/biom12020268)
Supplement: Supplementary file 1 [file biomolecules-12-00268-s001.zip › biomolecules-1545339-supplementary.pdf]

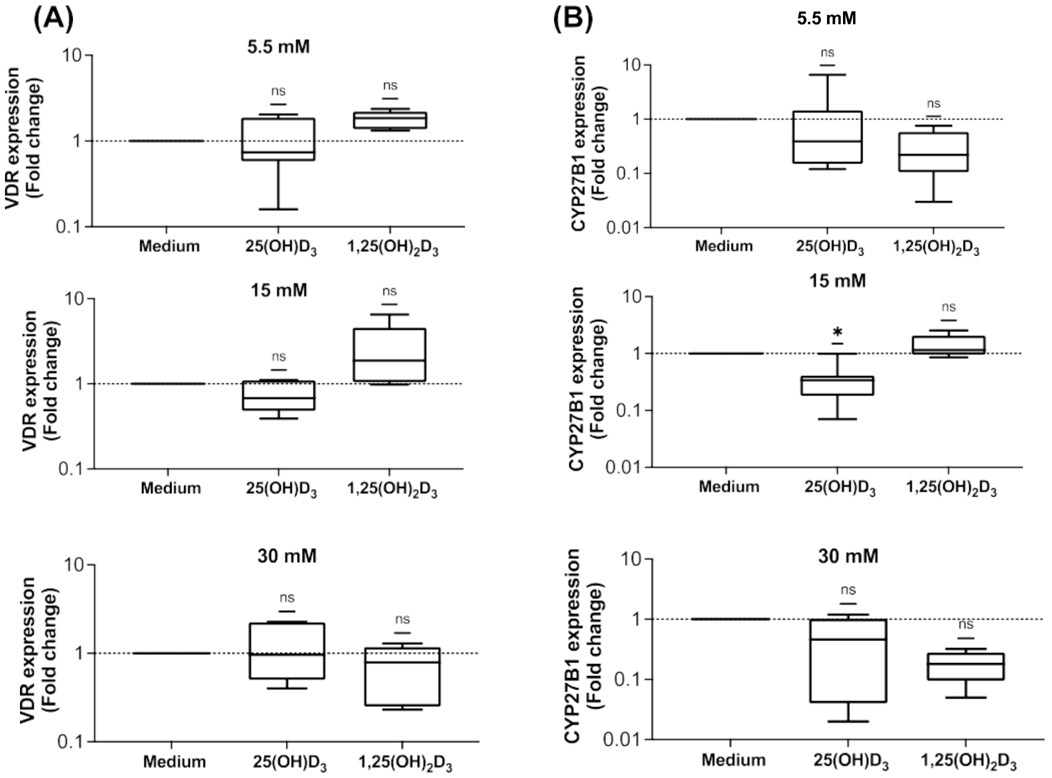

Figure S1. Effect of vitamin D on VDR and CYP27B1 gene expression in infected macrophages. Macrophages were cultured for 24 h in different glucose concentrations (5.5, 15, or 30 mM) and then infected with *M. tuberculosis* H37Ra at MOI 1. Macrophages were supplemented with 25(OH)D_3 or 1,25$(OH)_2D_3$ (10 nM or 1 µM) and incubated for an additional 24 h. The supernatants were removed, and the macrophages lysed for total RNA extraction, followed by cDNA synthesis rand VDR and CYP27B1 gene expression analyzed by qPCR. Individual results of (A) VDR and (B) CYP27B1 gene expression are reported as fold change relative to the medium. * $p<0.01$, Medium vs. 25(OH)D_3, Wilcoxon test.

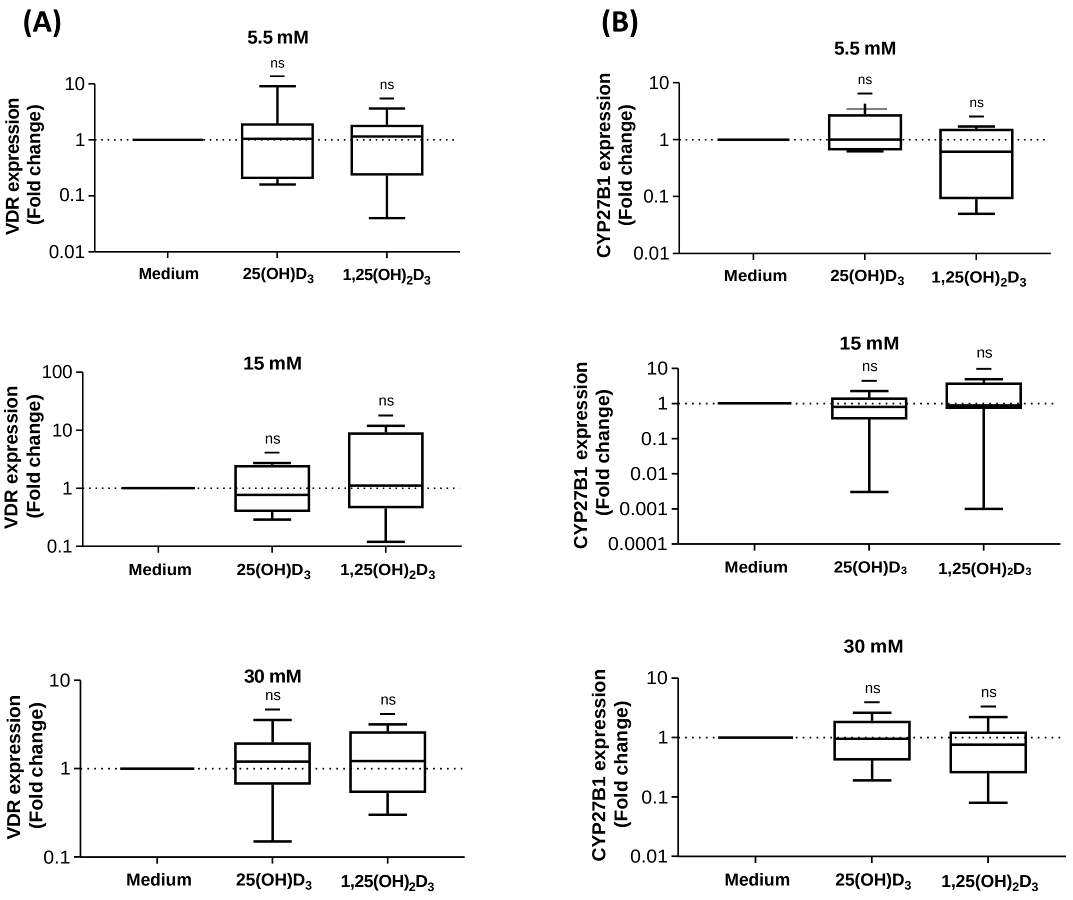

Figure S2. Effect of vitamin D on VDR and CYP27B1 gene expression in uninfected macrophages. Macrophages were cultured for 24 h in different glucose concentrations (5.5, 15, or 30 mM). Cells were supplemented with $25(OH)D_3$ or $1,25(OH)_2D_3$ (10 nM or 1 μM) and incubated for an additional 24 h. The supernatants were removed, and the macrophages lysed for total RNA extraction, followed for CDNA synthesis and VDR and CYP27B1 gene expression analysis by qPCR. Individual results of VDR (A) and CYP27B1 (B) gene expression are reported as fold change relative to the medium.

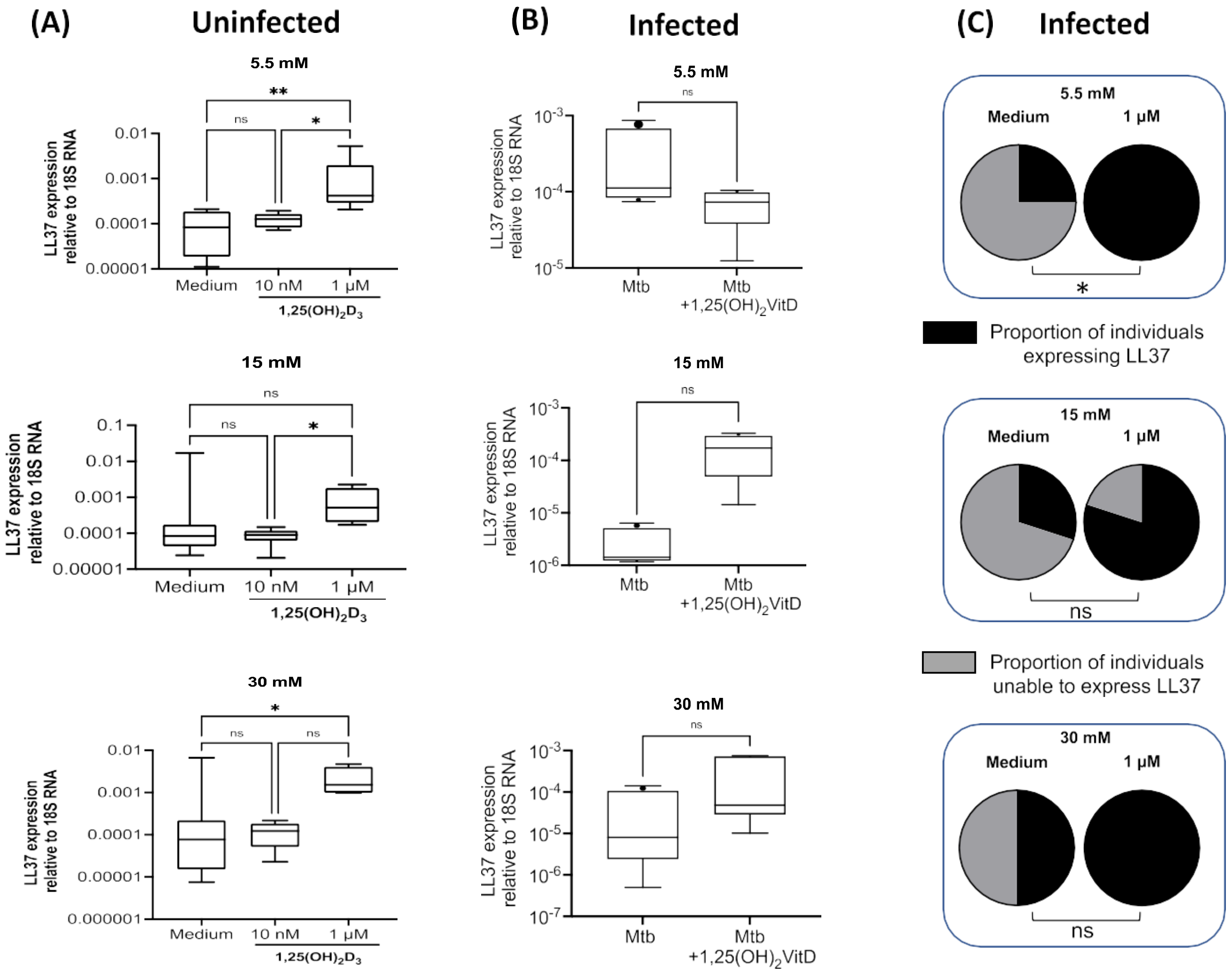

Figure S3. 1,25(OH)2D3 increases LL37 gene expression in infected macrophages. Macrophages were cultured for 24 h in different glucose concentrations (5.5 mM, 15 mM, or 30 mM), infected with *M. tuberculosis* at MOI 1, supplemented with 1,25(OH)2D3 (10 nM or 1 μM), and incubated for additional 24 h. The macrophages were lysed, and the total RNA was purified and used for cDNA synthesis. LL37 gene expression was measured by qPCR and reported as relative to 18s RNA. LL37 gene expression in uninfected macrophages (A), infected macrophages and (C) proportion of individuals expressing and unable to express LL37. * $p<0.01$, Fisher's exact test for categorical variables. Depicted are box plots with median and quartiles. * $p<0.01$, ** $p<0.001$, Medium vs. 25(OH)D3 (10 nM or 1μM), Kruskal-Wallis' nonparametric ANOVA, followed by Dunn's post-test.

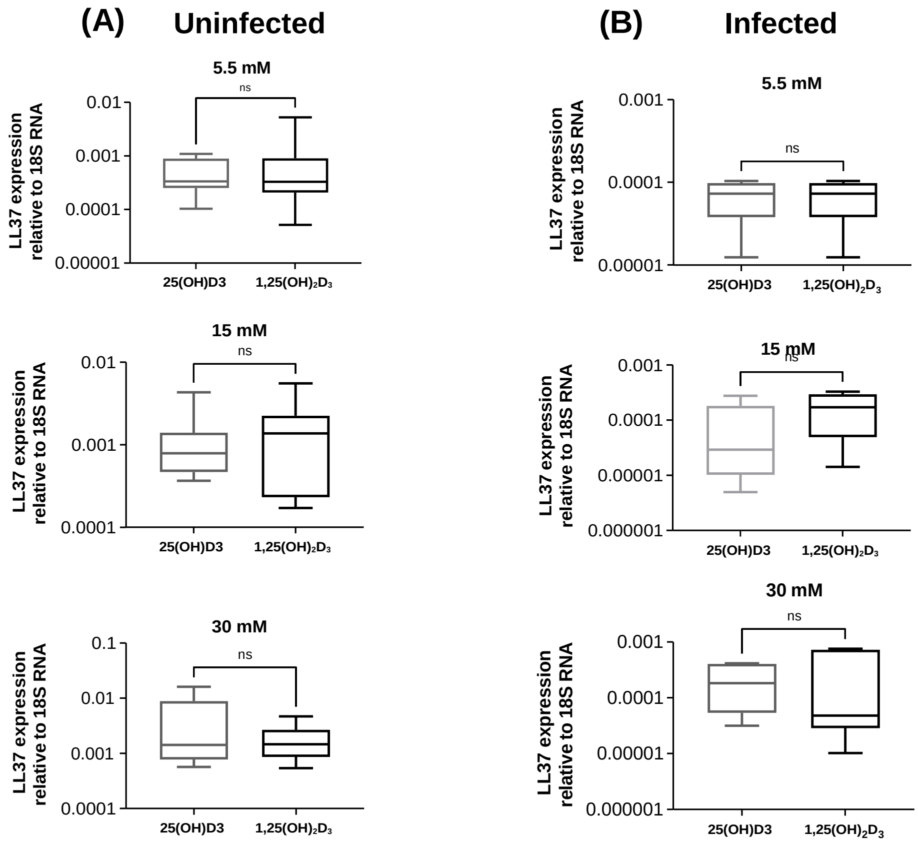

Figure S4. $25(OH)D_3$ and $1,25(OH)_2D_3$ equally increase LL37 gene expression in macrophages independently of the glucose concentration. Macrophages were cultured for 24 h in different glucose concentration (5.5, 15, or 30 mM), infected with *M. tuberculosis* at MOI 1, and supplemented with 1 µM of $25(OH)D_3$ or $1,25(OH)_2D_3$, and incubated for additional 24 h. The macrophages were lysed, and the total RNA was purified and used for cDNA synthesis. LL37 gene expression was measured by qPCR and reported as relative to 18S RNA in uninfected macrophages (A) and infected macrophages (B).
